# Neuron Loss in Transgenic Mouse Models of Alzheimer's Disease

**DOI:** 10.4061/2010/723782

**Published:** 2010-08-12

**Authors:** Oliver Wirths, Thomas A. Bayer

**Affiliations:** Division of Molecular Psychiatry and Alzheimer Ph.D. Graduate School, Department of Psychiatry, University of Goettingen, von-Siebold-Str. 5, 37075 Goettingen, Germany

## Abstract

Since their initial generation in the mid 1990s, transgenic mouse models of Alzheimers's disease (AD) have been proven to be valuable model systems which are indispensable for modern AD research. Whereas most of these models are characterized by extensive amyloid plaque pathology, inflammatory changes and often behavioral deficits, modeling of neuron loss was much less successful. The present paper discusses the current achievements of modeling neuron loss in transgenic mouse models based on APP/A*β* and Tau overexpression and provides an overview of currently available AD mouse models showing these pathological alterations.

## 1. Introduction

Alzheimer's disease (AD) represents the most frequent form of dementia and is characterized by two major neuropathological hallmarks: (i) extracellular plaques composed of the 40–42 residues A*β* peptide [1] and (ii) neurofibrillary tangles (NFTs), consisting of abnormal phosphorylated Tau protein [2]. There is increasing evidence that, in addition to the well-known extracellular amyloid deposition in the parenchyma, A*β* peptides accumulate within neurons [3]. It has been hypothesized that this initial accumulation is one of the earliest pathological events, which is able to trigger the cascade leading to neurodegeneration [4]. Whereas the vast majority of AD cases occur sporadically, a small percentage (<2%) of all cases represents familial forms of AD with an autosomal dominant mode of inheritance. Identification of the underlying mutations opened manifold opportunities for the generation of transgenic mouse models. Since their initial generation in the mid 1990s, transgenic mice have been proven to represent valuable model systems reflecting various pathological aspects of AD including plaque deposition, inflammatory changes or behavioral abnormalities (reviewed in [5, 6]). In the present short paper, we summarize the current achievements of modeling neuron loss in transgenic mice based on APP/A*β* overexpression.

## 2. APP-/A*β*-Based Mouse Models with Neuron Loss

A variety of different transgenic AD mouse models have been developed during the last 15 years which can be categorized as either APP single transgenic mice (e.g., PD-APP [7], Tg2576 [8], APP/Ld [9], TgCRND8 [10], APP23 [11], tg APP_ArcSwe [12], APP-Au [13], or APPE_693Δ_ [14]), bigenic mice expressing both APP and PS1/PS2 or Tau (e.g., APPswe/PS1dE9 [15], APP/PS1 [16], PS2APP [17], APP/PS1KI [18], or APP/tau [19]), and triple transgenic mice expressing APP, PS1, and Tau (e.g., 3xTg [20] or TauPS2APP [21]). Whereas most of these models present abundant extracellular amyloid plaque pathology, several efforts modelling significant neuron loss remained less successful [22, 23]. 

First evidence for neurotoxic *in vivo* properties of A*β* came from a transgenic mouse model expressing murine A*β* under the control of the mouse Neurofilament-light gene (NF-L) promoter, ensuring neuronal expression. This resulted in abundant neurodegeneration, with biochemical and morphological evidence for an apoptotic mechanism [24]. Later, a transgenic mouse model expressing human APP with the Swedish mutation (APP23) under the control of the murine Thy1-promoter was reported, showing significant hippocampal CA1 neuron loss (−14%) at the age of 14 to 18 months. These mice show an age-dependent extracellular plaque deposition primarily in neocortex and hippocampus, accompanied by severe gliosis. The number of CA1 neurons is inversely correlated with CA1 plaque load and neuron loss was observed primarily in the vicinity of extracellular plaques [25]. It has been shown that focal neuronal toxicity is associated with extracellular A*β* deposits when they occur in a fibrillar beta-pleated sheet confirmation [26]. Surprisingly, no differences in the neocortical synaptic bouton number as well as in synaptophysin protein levels were detected during aging or in comparison with age-matched nontransgenic control mice [27].

Analysis of 17-month-old APP_751SL_/PS1M146L transgenic mice using unbiased stereologic methods revealed a loss of CA1-3 neurons in a magnitude of ~30% compared to age-matched PS1 control animals. Interestingly, the plaque load was approximately 10% smaller than the level of hippocampal pyramidal cell loss in these mice, indicating a loss of neurons at sites of A*β* aggregation but also clearly observed in areas distant from extracellular A*β* deposits. This observation points to the potential involvement of more than one mechanism in hippocampal neuron loss in this mouse model [29]. A quantitative study of synaptophysin-immunoreactive presynaptic boutons (SIPBs) revealed an age-related loss in both APP_751SL_ and PS1M146L single transgenic mice within the stratum radiatum, which was most severe in APP751SL/PS1M146L mice extending also to plaque-free regions [30].

**Figure 1:**
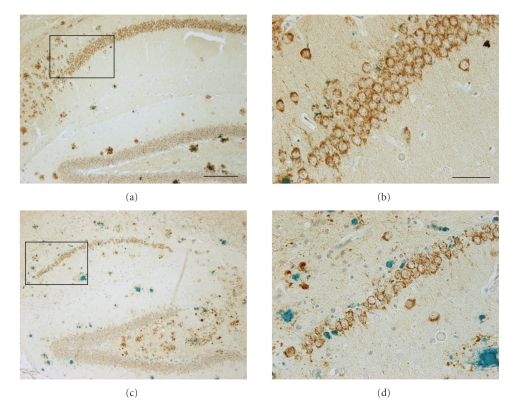
Hippocampal neuron loss in the APP/PS1KI mouse model of AD. (a) APP (brown) and A*β* staining (green) in the hippocampal formation of a 2-month-old and (c) a 10-month-old APP/PS1KI mouse. (b,d) Higher magnification of the CA1 granular cell layer of a 2-month-old (b) and a 10-month-old APP/PS1KI reveals profound neuron loss at the later time point. Scale bars: (a,c): 200 *μ*m, (b,d): 50 *μ*m.

Another model showing a more severe hippocampal neuron loss is the APP/PS1KI mouse model [18]. At the age of 10 months an extensive neuron loss (>50%) in the hippocampus was reported, that correlated with the accumulation of intraneuronal A*β* and Thioflavin-S positive intracellular material [18] (Figure 1). Extending these studies to earlier ones revealed that this CA1 neuron loss is already detectable at the age of 6 months. At this time point, a loss of 33% of CA1 pyramidal neurons compared to PS1KI littermates could be demonstrated, together with a decrease in the CA1 volume (−30%), an atrophy of the entire hippocampus of 18% and synaptic alterations, including reduced levels of pre- (SNAP25, clathrin light chain) and post-synaptic markers (PSD-95). In addition, recordings of field excitatory postsynaptic potentials (fEPSPs) revealed a significant reduction of 6 months in APP/PS1KI compared to PS1KI or nontransgenic mice [31]. A detailed stereological comparison of neuronal numbers in frontal cortex and thalamus, representing brain areas with intra- and extracellular A*β* accumulation (frontal cortex) or with only extracellular A*β* pathology (thalamus), revealed an early loss of cortical neurons starting at the age of 6 months. This neuronal loss correlated with the transient intraneuronal A*β* accumulation. No neuron loss could be observed in the thalamus where on extracellular A*β* plaques, however, in a comparable amount as in the cortex were present [32]. A related observation was made in distinct cholinergic brain stem nuclei (Mo5, 7N) in this mouse model, where neuronal loss at 6 or 12 months of age correlated with the presence of intraneuronal A*β* peptides [33]. Interestingly, a significant loss of parvalbumin- (PV)-positive interneurons in CA1-2 (40%–50%) and calretinin- (CR)-immunoreactive interneurons in the hilus and dentate gyrus (37%–52%) has been recently reported in 10-month-old APP/PS1KI mice [34]. This is in the range of PV- and CR-positive interneuron losses in the dentate gyrus of postmortem brain specimen from AD patients. In addition, a significant neuron loss has been found in the granule cell layer of the dentate gyrus of 12-month-old APP/PS1KI mice, where abundant A*β* deposition is present. This loss is likely due to local extracellular plaque pathology [26], in combination with a complete loss of neurogenesis already at the age of 6 months [35, 36], which prevents any re-integration of new-born neurons in that particular cell layer. 

A recently described mouse model expressing mutant APP and PS1 under the control of the murine Thy1 promoter (5XFAD mice) underscores the potential influence of intraneuronal A*β* accumulation on the loss of neurons. Analysis of cresyl violet stained sections in 9-month-old mice revealed a reduced number of cortical layer 5 neurons, a region with robust intracellular A*β* immunoreactivity. The same holds true for the subiculum where neurons where pale or entirely missing [28]. In a very recent report, cortical and hippocampal neuron numbers were analysed by design-based unbiased stereological methods in 12-month-old female mice, verifying the discrete layer 5 neuron loss. No reductions in neuron numbers and no intraneuronal A*β* immunoreactivity were detected in the CA1 layer of the hippocampus adding further evidence to the assumption that intraneuronal A*β* accumulation is closely associated with neuron loss [37]. These mice also show synaptic alterations demonstrated by a decline in synaptophysin levels already at 4 months of age as well as significantly reduced syntaxin and PSD-95 levels at the age of 9 months [28].

Besides full-length A*β* peptides ending at amino acid 40 or 42, N-terminally truncated peptides have recently gained in importance. One of the most abundant truncated peptides in AD brain is *A* *β*_pE3–42_ carrying a pyroglutamate (pE) at position 3 [38]. It has been demonstrated that this peptide is characterized by a higher aggregation propensity [39], stability [40], and increased toxicity compared to full-length A*β* [41]. Recently *in vivo* toxicity of this peptide has been demonstrated in a mouse model expressing *A* *β*_pE3–42_ in neurons under the control of the murine Thy1 promoter. Glutamate (E) at position three of A*β* has been mutated into glutamine (Q), as it is well established that glutamine becomes much faster converted into pyroglutamate. These mice showed a severe neurological phenotype with premature death and abundant loss of cerebellar Purkinje cells [42].

Very recently, a new transgenic mouse model expressing human APP with the APP_E693Δ_ mutation has been published [14]. This mutation has been initially described in a Japanese pedigree showing Alzheimer's-type dementia and is characterized by decreased total A*β* secretion but increased resistance to proteolytic degradation and enhanced oligomerization [43]. The resulting transgenic mice displayed intraneuronal accumulation of Ab oligomers staring at the age of 8 months, however, no extracellular A*β* plaque formation could be detected even at the age of 24 months. In addition, micro-and astroglial accumulation was observed, as well as a significant decrease in the number of NeuN-positive cells in the hippocampal CA3 region at 24 months of age compared to age-matched nontransgenic littermates and transgenic mice expressing wild-type APP. Furthermore, an age-dependent decrease in synaptophysin levels was shown by means of immunohistochemistry starting at the age of 8 months, which coincides with impairments in synaptic plasticity as shown by *in vivo* electrophysiology [14] (see Table 1).

## 3. APP and Tau Transgenic Mouse Models

Recently a triple transgenic mouse model has been developed expressing both mutant APP (Swedish) and Tau (P301L) on a mutant PS1 knockin background (3xTg-AD mice) [20]. Intracellular A*β* is apparent between 3 and 4 months in these mice and precedes the deposition of extracellular A*β* peptides starting around the age of 6 months. At this time point synaptic plasticity was already strongly compromised in these mice, as shown by impaired long-term potentiation [20]. Behavioral analyses have suggested that intracellular A*β* accumulation is functionally linked to cognitive impairment in these mice, as they develop deficits in long-term retention at the age of 4 months, a time point prior to plaque deposition where only intracellular A*β* is present [44]. Morphological alterations of hippocampal synapses have been characterized in 13-month-old 3xTg-AD mice and age-matched PS1KI control mice. The numeric density of synapses, the average synaptic contact area as well as the synaptic surface density were not altered, however, 3xTg-AD mice showed a significant reduction in the fraction of perforated synapses, which is believed to represent a reliable indirect index of synaptic plasticity [45]. 

A double transgenic mouse line based on Tg2576 mice crossed with VLW mice (Tau G272V, P310L, R406W) showed increased amyloid deposition at the age of 16 months compared to APP single transgenic mice. In addition, APP/Tau mice revealed a significantly reduced neuron number in the entorhinal cortex at 9 months of age compared to APP single transgenic, Tau single transgenic, or wild-type mice, which were extended to the CA1 layer at the age of 16 months. It was further reported that cell death in these APP/Tau mice preceded overt amyloid plaque formation and NFT formation and did not correlate with amyloid burden in any of the regions examined [19].

Another new transgenic mouse line expressing mutant APP, Presenilin-2 (PS2), and Tau (TauPs2APP) has been recently described. It has been demonstrated that A*β* in these triple transgenic mice impacts on Tau pathology by increasing the phosphorylation of Tau at serine 422. However, despite of increased levels of phosphorylated Tau, no differences in neuron numbers were detected in hippocampal subregions comparing triple transgenic and wild-type mice. Quantitative receptor autoradiography revealed significantly reduced mGluR2 levels in aged triple transgenic mice, which were however not different from the PS2APP double transgenic control line, arguing against a prominent role of increased Tau phosphorylation [46].

## 4. Conclusion

In summary, there is no doubt that transgenic mice have been proven to be valuable model systems in modern AD research. There is accumulating evidence that significant neuron loss in APP/A*β*-related transgenic mice is linked to intraneuronal A*β* accumulation, as this pathological alteration precedes neurodegeneration in almost all the models where neuronal loss has been convincingly reported. If this is also true for the human situation is currently less clear, as intraneuronal A*β* seems to be a transient phenomenon which might not be adequately detected in end-stage AD patients. Although it has been shown that reductions in Tau levels prevented behavioral deficits in transgenic mice expressing human amyloid precursor protein and also protected both transgenic and nontransgenic mice against excitotoxicity [47], overexpression of mutant Tau in APP or APP/PS transgenic mice does not result in dramatic effects on neurodegeneration. One possibility might be that murine neurons could be devoid of the downstream pathways necessary for A*β*-induced toxicity leading to tau aggregation in NFTs in human AD brain.

## Figures and Tables

**Table 1:** Overview of transgenic AD mouse models in which neuronal loss and/or intraneuronal A*β* accumulation has been reported. In addition, information on the transgene, extracellular plaque onset, and intraneuronal A*β* accumulation are given. (n.d.: not determined).

Transgenic mouse model	Mutation APP	Mutation PS1	Promoter	Plaque onset	Intraneur. A*β*	Neuron loss	Synaptic dysfunction	Reference
NF-L-A*β*	—	—	NF-L (A*β*)	n.d.	√	√	n.d.	[24]
APP23	Swedish	—	Thy1 (APP)	6 m	n.d.	14–18 m	—	[25]
APP_751SL_x PS1M146L	Swedish, London	M146L	Thy1 (APP) HMG-CoA (PS1)	3 m	√	17 m	√	[16]
APP_751SL_/ PS1KI	Swedish, London	M233T, L235P	Thy1 (APP) PS1 knockin	2 m	√	6 m	√	[18]
5XFAD	Swedish, Florida, London	M146L, L286V	Thy1 (APP, PS1)	2 m	√	9 m	√	[28]
TBA2	—	—	Thy1 (AbpE_3-42_)	2 m	√	2 m	n.d.	[42]
APP_E693Δ_	Japanese	—	Prp (APP)	—	√	24 m	√	[14]
3xTg	Swedish	M146V	Thy1 (APP, Tau) PS1 knockin	6 m	√	n.d.	√	[20]
APP/tau	Swedish	—	Prion Protein (APP) Thy1 (Tau)	9 m	n.d.	9 m	n.d.	[19]
TauPS2APP	Swedish	N141I (PS2)	Thy1 (APP, PS2, Tau)	4 m	n.d.	—	n.d.	[46]
